# Book Review

**Published:** 1935

**Authors:** 


### Reviews of Books

**Hugh Owen Thomas: his Principles and Practice.** By David McCrae Aitken, M.B., Ch.B., F.R.C.S. Pp. x., 96. Illustrated. London: Oxford University Press. 1935. Price 12s. 6d.—This is a companion volume to the book describing the life and personality of Hugh Owen Thomas. It gives an account of the evolution of many splints and mechanical apparatus, invented and made by the ingenious brain and hands of this brilliant man. There are eight plates, five illustrating various splints; one a picture of their inventor; one showing the result and value of prolonged complete rest as taught by Mr. Thomas, illustrated by a case of the author's, and one amusing picture, showing the correct position for inflamed joints (probably gout) published in 1782. The principles taught by Mr. Thomas and now followed and enlarged by Dr. Böhler of Vienna in his recently published book are clearly explained by one who has evidently thoroughly imbibed and practised them. There is a complete list of H. O. Thomas's writings. The book is well printed and of a convenient size, and there is a good index.

**The "Albatross": being the Biography of Conrad Fitz-Gerald, M.R.C.S.** By C. T. Fitz-Gerald. Pp. 208. Illustrated. Bristol: J. W. Arrowsmith Ltd. 1935. Price 8s.—This enigmatic title disguises the biography of the Senior Alumnus of the Bristol Medical School, a biography that every member of the school ought certainly to possess. The quite extraordinarily interesting life of Conrad Fitz-Gerald is given us here in detail. There is a good description of the life of a medical student in the days when one was bound apprentice to a surgeon or an apothecary, hospital training following 'prentice days. Next come accounts of early voyages as ship's surgeon, both in sail and steam. Then the chance to settle in very primitive Newfoundland, where Fitz-Gerald conducted for sixty years a practice of the most varied and unexpected sort over an area of some forty miles by fifty, much of the travelling being by sea or on snowshoes. In the early days anæsthetics were regarded as unnecessary—just as well, since they were usually unobtainable (by the way, the author is quite wrong about the introduction of cocaine). Every type of case is recorded ; burning, drowning, frost-bite, wolf-bite, broken limbs, epidemics—especially of diphtheria, particularly dreaded and deadly in the pre-antiserum days. Much of the book consists of extracts from the log of the *Albatross*, the good doctor's yacht, where the most terrific fights with wind, storm and flood are recorded in the truly laconic manner ; receiving as a rule less mention than the bags of trout, partridge, deer and silver fox, for the doctor was and is a keen sportsman. The book has been written for lay readers, but there is no medical who will not thoroughly enjoy it. The publishers have brought out a very pleasant, handy and well illustrated biography. All good wishes to the hero !

**Diabetes Mellitus.** By T. H. OLIVER, M.D., F.R.C.P. Pp. vi., 120. London : John Bale, Sons & Danielsson. 1935. Price 3s. 6d.—This is an excellent little monograph upon Glycosuria. This is definitely a contribution, and a very thought-provoking one, to the whole question. The author has been very much struck by the great differences to be found in the symptomatology, and reaction to treatment with insulin which are met with in cases which all present themselves with sugar in the urine. The young diabetic who goes off with ketosis, the older one who gets obese, the rare cases which are resistant to insulin have all given him furiously to think. And as a result of his clinical observations he has evolved a theory as to the causation of the different classes which will well repay careful study. He then goes on to a brief but very practical review of the treatment for the different classes and of the complications which may arise. Perhaps everyone will not agree with all the suggestions. For instance, one of the possible diagnostic differentiations between the coma of ketosis and of hypoglycaemia is said to be that sometimes a softness of the eyeball may help to show that the case is one of hypoglycaemia, whereas the converse is generally stated in text-books. Few books actually find their way into the reviewer's own shelves. This one certainly must.

**Modern Treatment in General Practice.** Edited by C. P. G. WAKELEY, F.R.C.S. Volume 1 (Second Edition), pp. viii., 426; Volume 2, pp. viii., 382. Illustrated. London: Baillière, Tindall & Cox. 1935. Price 10s. 6d. each volume.—A series of articles on "Modern Treatment in General Practice" which have appeared during the last year or two in the *Medical Press and Circular*, has been published in two volumes under the general editorship of Mr. C. P. G. Wakeley. The plan adopted by the various authors is admirable, because each article begins with a brief clinical description of the disease or condition dealt with. Thus the indications for treatment and the remedies available are brought closely together. The articles are up to date, but old and tried methods are included as well as new. One most interesting chapter is that on the treatment of early syphilis (volume 1, p. 38). The author's conclusion is important: "although modern methods have undoubtedly proved a great advance on the older forms of treatment they do not yet provide us with a certain means of rapid cure." Dr. Allison is to be congratulated on the amount of information he has compressed into his admirable chapter on "Disseminated Sclerosis." On the whole he seems to regard the administration of Vitamin A as the most hopeful line to adopt. Dr. Mapother's contribution on "Sudden Insanity" is full of helpful advice to general practitioners and may lead to a more extensive use of the clauses of the Mental Treatment Act 1930, permitting the acceptance of "voluntary" and "temporary" patients as well as "certified." E. Watson-Williams has written an excellent chapter on "Otitis Media in Children," in which the instructions as to treatment are most precise. His methods are such as have been shown by experience to be efficacious. It is interesting to note that he is not a very ardent advocate of myringotomy; "there is some evidence," he writes, "that the incision made artificially heals more rapidly and with less risk to hearing than a natural perforation of the membrane." The powdered magnesium sulphate treatment which he recommends at a certain stage is undoubtedly a great therapeutic advance. One might venture on a mild criticism that some diseases receive almost an undue share of attention—as an instance, one may quote separate chapters on "The Treatment of Rheumatoid Arthritis in General Practice," "The Spa Treatment of Chronic Rheumatism" and "A Practical Survey of Spa Facilities." In the last named there is surely a misprint on page 365 (volume 1): "Llanwetyd"

seems to appear in place of that extraordinarily beautiful spot Llanwrtyd Wells, a spa which deserves wider recognition on account of the strength of its sulphur spring. Dr. Neale has given a good description of "Pink Disease and its Treatment," but the illustrations fail to depict the outstanding characteristics of the disease. Some really good colour photographs are needed to portray pink disease in recognisable form. Speaking of the chapters on children's diseases in general they are deserving of the highest praise. Mr. William Anderson on "The Rôle of Surgery in the Treatment of Pulmonary Tuberculosis," directs attention to the great progress made in thoracic surgery in recent years. In spite of Macewen's pioneer work this branch of surgery has not received the attention it deserves in Great Britain. Anderson's article, in spite of its brevity, is full of sound and practical ideas. Price Thomas on "Empyema" is not quite so helpful. He makes no mention of the plan of closing an empyema after evacuating the pus, but seems to advocate drainage in all circumstances. He deprecates indiscriminate needling in search of an empyema, but does not go the whole hog and say that needling should not be resorted to unless everything is prepared for aspiration or surgical drainage should pus be found. Also he still thinks that blowing and sucking from Wolff's bottles will re-expand the collapsed lung rather than over-distend the sound one. Hamilton Bailey is exceedingly good on "Acute Retention of Urine." So, too, is Meurice Sinclair on "The Thomas Splint." Whilst these two volumes cannot, of course, cover the whole field of treatment in general practice, it can safely be said that no subject is dealt with which may not confront any general practitioner, and dealt with in such a way as to offer fresh ideas and up-to-date methods. This collection of essays is worth keeping at hand for reading at leisure as well as for quick reference in emergencies.

**Oxygen and Carbon Dioxide Therapy.** By A. CAMPBELL, M.D., D.Sc., and E. P. POULTON, D.M. Pp. xv., 179. Illustrated. London: Oxford University Press. 1934. Price 12s. 6d.—In this book the authors have supplied a want long felt by the Medical Profession, they have succeeded in furnishing the reader with an up-to-date survey of the whole subject based upon the results of experimental research and a very large number of clinical observations. The clinician will find much information of great value in determining for which class of case this form of treatment is likely to be suitable. The statistics show that carbon dioxide should not be administered where there are indications of heart failure, and that oxygen is not indicated in the breathlessness of uræmia or diabetes. But the authors point out the futility of statistics compiled from the results yielded by haphazard methods of administration, and the favourable reports of the treatment of pneumonia by oxygen, in the early stages, refer to those cases in which the oxygen tent has been employed. In the section on physiological and pathological considerations are to be found, in condensed but intelligible form, the description of methods used in estimating gas tensions and the definitions of many clinical conditions such as the various types of anoxæmia—a clear conception of which is necessary for the understanding of the physio-chemistry on which the work is based. No trouble has been spared in collecting evidence and in reviewing the published works of the most reliable experimenters, both academical and clinical, and students of this important subject will find in the bibliography a unique table of references. The readability of this useful volume is enhanced by its convenient arrangement and by the excellent style in which it has been produced by the Oxford University Press.

**Forensic Medicine.** By Douglas J. A. Kerr, M.D., F.R.C.P. Pp. x., 311. Illustrated. London: A. and C. Black Ltd. 1935. Price 15s.—There are already a number of text-books devoted to Forensic Medicine, so Dr. Kerr shows considerable courage in adding to this list. He is, however, a lecturer on this subject, as well as a police surgeon, and therefore well equipped for his task. The illustrations both of murdered bodies and suicides are graphic, not to say lurid, but one wonders if they are worth the expense involved in printing them. The chapter on wounds and wounding also includes the liability of a doctor for negligence and malpractice. We think this would have been better dealt with in a separate chapter, devoted to his relations and obligations to the public. The general layout of the book is good, and the text is well written and lucid. On the whole we think this volume fills a gap in the literature of this subject, and so we have no hesitation in recommending it both to practitioners and students.

**Angina Pectoris and its Surgical Treatment.** By RENE LERICHE, F.R.C.S., F.A.S.A. Pp. 24. Illustrated. Glasgow: Jackson, Wylie & Co. 1935. Price 1s. 6d.—This is really the report of a lecture rather than a book. It is very interesting, and may perhaps prove to mark a new era in the treatment of angina. The author shows by experimental observation on animals that the coronary arteries are dilated by cutting off sympathetic nervous impulses. Ligature of a coronary artery may be quickly fatal from ventricular fibrillation, or necrosis and perforation of the ventricular wall, but if the immediate dangers are survived the anastomotic circulation soon opens up and the heart wall recovers. Removal of the stellate ganglion, on one or both sides, is therefore a physiologically sound procedure. It cuts off the painful sensory impulses, and opens up the collateral circulation. His clinical experience shows that the operation is not dangerous to life, and will relieve the attacks of angina if the quality of the heart muscle is not hopelessly impaired.

**Ophthalmology in General Practice.** By O. G. MORGAN. Pp. vi.,59. Illustrated. London: John Bale, Sons and Danielsson, Ltd. 1935. Price 2s. 6d.—This small book, one of a series of Pocket Monographs on Practical Medicine, is designed to assist the General Practitioner in dealing with those ocular conditions frequently recurring in private practice, and with such less common ones as require, for the safety of the eye, early and accurate diagnosis. It is natural, therefore, that the major portion of the book should be taken up with methods of examination and with inflammatory conditions, but there are useful sections on glaucoma, fundus conditions, squint and errors of refraction. Within its limited scope the book conveys a large amount of information in an essentially practical manner. It is easy to read, and is well worth the very modest sum required for its purchase.

**Handbook of Anæsthetics.** Fourth Edition. By J. STUART ROSS, M.B., Ch.B., F.R.C.S., and H. P. FAIRLIE, M.D. Pp. 299. Illustrated. Edinburgh: E. and S. Livingstone. 1935. Price 10s. 6d.—This popular work on Anæsthesia has proved itself well worthy of revision, and Dr. Fairlie has produced the present—fourth—edition without deviating from the original scheme of presenting the reader mainly with first principles upon which he may build up his own particular methods. On the whole it has not been necessary to make many radical alterations owing to the soundness of the earlier issues—naturally a new chapter has had to be added with an account of the more commonly used basal narcotics, which should be helpful to the student when selecting the drug suitable for each particular case. In a few instances the details of technique seem to us inadequate, for example in the description of the introduction of the intratracheal catheter by the nasal route, but the outstanding feature of the book is the vivid manner in which various clinical phenomena are presented. And this, combined with the author's talent for analysing and interpreting the signs of anæsthesia make this book a valuable and safe guide to administration. Among the additions we notice an account of Borchardt's apparatus and technique for administering ethyl chloride in mouth operations, also Minnitt's nitrous oxide apparatus for use in labour. We are not altogether surprised to see the suggestion that the safety of chloroform capsules for midwifery may be open to question. The book is small but complete and is exceptionally readable, and has been produced in excellent style.

**The Theory and Practice of Anæsthesia.** By M. D. NOSWORTHY, M.D. Pp. 223. Illustrated. London: Messrs. Hutchinson & Co. 1935. Price 12s. 6d.—Dr. Nosworthy has devoted a great deal of thought to the teaching of students in the practice of administering anæsthetics, and, having acquired an insight into their difficulties, has been able in this book to cater for their needs with exceptional clearness. Yet he has not made the work too rudimentary, with the result that even experienced anæsthetists will find much of interest in these pages. Owing to the importance of a knowledge of the scientific principles governing the art of administration the author has included in the initial chapters an account of the more important and generally accepted data bearing upon the subject of inhalation anæsthetics, with special reference to the causation of acidosis, the function of oxygen and of carbon dioxide, the physiology of rebreathing and the development and prevention of shock, each of which topics, he insists, deserves careful study. A good deal of prominence is given to chloroform and ether because the author considers that the successful employment of these drugs equips the student with a satisfactory groundwork of knowledge from which he may safely turn to more modern and intricate methods. Nitrous oxide is fully described with its advantages and limitations and with special reference to the method of secondary saturation described and practised by McKesson, a proceeding which, in the author's opinion, puts an unnecessary strain on the circulation even when administered by the most approved apparatus. A welcome feature of this little work is the concise but comprehensive review of the various methods of premedication with suggestions as to the choice of those most suitable in various cases. The references at the end of each chapter are full, and should be useful to those who wish to make further study of the topics concerned. We welcome this addition to the literature on anæsthesia and congratulate the author on the book's convenient arrangement and on the inclusion of a carefully compiled index. The printing and general style are excellent.

**Medicine for Nurses.** By W. GORDON SEARS, M.D., M.R.C.P. Pp. viii., 412. Illustrated. London: Edward Arnold & Co. 1935. Price 8s. 6d.—In this text-book the subjects are clearly and methodically arranged, emphasis being placed on the practical side of medical work. The diagrams employed are particularly helpful, as also are derivations of words which would otherwise often appear meaningless. The diseases of the blood are described in a manner which will be new to many nurses, but scientific details are explained as simply as possible, and so enhance the interest of the subject. The book should be a valuable addition to the Nurses' Library, and should prove most useful to those assisting in teaching student nurses. The printing is good and the general "lay-out" very pleasing.

**Theory and Practice of Nursing.** Fourth Edition. By M. A. GULLAN. Pp. xvi., 258. Illustrated. London: H. K. Lewis & Co. Ltd. 1935. Price 9s. net.—This text-book, written for the instruction of nurses, should be very useful to the sister-tutor as a groundwork for her own lectures. It contains extremely good chapters on dietetics and digestion with most interesting diagrams of metabolism. The instructions on practical nursing and methods of applying treatment are very valuable, and there are excellent chapters on the nursing of special conditions such as heart and lung diseases, whilst the section on private nursing should be studied and digested by every private nurse. The book is well got up and nicely bound. If it has any fault it is perhaps a trifle dull at first sight, and some of the technical terms used, such as "fastigium" and "exanthem," seem rather unnecessary.

**The Nursing and Diseases of Sick Children.** Second Edition. Edited by A. MONCRIEFF, M.D., F.R.C.P. Pp. xviii., 583. Illustrated. London: H. K. Lewis & Co. Ltd. 1935. Price 15s.—In this book, which emanates from the Nursing School of the Hospital for Sick Children, Great Ormond Street, the practice, so popular in current medical text-books, of having different sections written by special authors has been followed with very happy results. The accuracy of the nursing details has been further ensured by their supervision by the sister-tutor, and it may be taken that the book reflects accurately the nursing methods of Great Ormond Street. The second edition has been completely revised and several new illustrations added. The latter, especially Mr. Keogh's clear and simple drawings, are a noteworthy feature. There is a very complete and accurate index and the type is clear and pleasant to read. This volume not only provides a complete text-book for nurses working for the examination for the Certificate for Sick Children's Nurses of the General Nursing Council, but is a work of reference which no nurse interested in sick children can afford to be without.

**The Medical Annual General Index, 1925-34.** Pp. xvi., 450. Bristol: John Wright & Sons Ltd. 1935. Price 12s. 6d.—Nearly all doctors who read anything at all take in *The Medical Annual*, and have done so for years, if only for the reason that it makes so much more reading, over a very wide field, unnecessary. But it is a tedious business to hunt through each of ten volumes for the information one wants, and this General Index will meet a real need. It is, however, much more than an index. Many of the suggestions made for better diagnosis and treatment in a year's literature, duly noticed in *The Medical Annual*, soon drop out of use. The present volume therefore supplies a very brief review, subject by subject, of those advances which seem likely to be of permanent value, and gives appropriate references to notices of the same during the past ten years. For instance, there is a six-page review of the Medical Diseases of Children and four pages on Diseases of the Respiratory Tract. In other words, here in a matter of fifty pages the practitioner's attention is called, by experts in their own line, to the really important advances in each branch of medicine and surgery for ten years past. If that does not attract the busy man it is difficult to think what will—nothing medical, we fear, unless, of course, he knows the ten volumes of the *Annual* by heart.

